# Dose-Intensity of Bisphosphonates and the Risk of Osteonecrosis of the Jaw in Osteoporosis Patients

**DOI:** 10.3389/fphar.2018.00796

**Published:** 2018-07-20

**Authors:** Sung-mok Jung, Sujeong Han, Hye-Young Kwon

**Affiliations:** ^1^Graduate School of Medicine, Hokkaido University, Sapporo, Japan; ^2^Department of Public Health Science, Graduate School of Public Health, Seoul National University, Seoul, South Korea; ^3^Division of Biology and Public Health, Mokwon University, Daejeon, South Korea

**Keywords:** bisphosphonates, osteonecrosis of the jaw, dose-intensity, population-based, National Health Insurance claim data

## Abstract

Objective: To examine the incidence rates and association between dose-intensity, stratified by exposure duration, of bisphosphonates and the risk of osteonecrosis of the jaw among Korean osteoporotic patients older than 50 years. Study Design and Setting: Using the population-based National Health Insurance Claims Data of Korea from January 1, 2006, through December 3, 2012, 13,730 new bisphosphonate users as of 2006 were identified. Truncated age-standardized incidence rate estimation and multivariate logistic regression analyses were conducted. Results: In this retrospective cohort study, increasing age-standardized incidence rates of ONJ attributed to bisphosphonate exposure were observed for individuals with less than 1 year, 1–2 years, over 2 years of defined daily dose (DDD) of bisphosphonate exposure (13.85, 16.19, and 38.20, respectively), using a truncated 2000 United States Standard Population. Also, over 2 years of bisphosphonate DDDs was associated with an increased risk of developing of ONJ with an adjusted odds ratio of 1.51 (95% confidence interval: 1.31–1.75), compared to individuals with less than 1 year of bisphosphonate exposure. Conclusion: Our data provided the evidence to support the association between risk of ONJ and duration of bisphosphonate exposure used in the treatment or prevention of osteoporosis.

## Introduction

Bisphosphonates are a part of standard pharmaceutical therapy for osteoporosis and for the prevention of osteoporotic fractures (Whitaker et al., 2012). As bisphosphonates can effectively improve bone density through the inhibition of osteoclast-mediated bone resorption (Huang et al., 2015), more than 190 million prescriptions of bisphosphonates are written annually worldwide (Ruggiero et al., 2009). However, bisphosphonates have well-known adverse reactions such as upper gastrointestinal irritation, which significantly affect compliance with medication among osteoporosis patients (Kennel and Drake, 2009). Osteonecrosis of the jaw (ONJ) is also a frequently occurring adverse reaction. Bisphosphonate-induced ONJ is a rare condition and the exact underlying pathophysiologic mechanisms remain unknown but may be related to altered bone remodeling or increased mineralization of local tissue (Lo et al., 2010). Since the first case of ONJ as a complication of treatment with high dose intravenous bisphosphonates was reported in 2003 (Migliorati et al., 2005), various studies have been conducted to assess the association between bisphosphonates and ONJ. Many studies have revealed that tooth extraction, cumulative dose and length of exposure to bisphosphonates seem to be the most important risk factors for developing bisphosphonate-associated ONJ (Fehm et al., 2009). Thus, there is the need to explore optimal duration of exposure to bisphosphonates among osteoporosis patients.

Nevertheless, although there is considerable controversy over the ideal duration of antiresorptive therapy, particularly since reports have emerged of atypical subtrochanteric fractures as well as ONJ during prolonged bisphosphonate therapy (Black et al., 2012), bisphosphonate-induced ONJ has not been sufficiently explored among Korean patients. In particular, the increasing risk of ONJ in proportion to the extent of exposure to bisphosphonates has not sufficiently been investigated in the Korean context (Lee et al., 2013; Kwon et al., 2015). Kwon et al. (2015) firstly demonstrated the association between ONJ and bisphosphonate administration among Korean osteoporotic patients using National Health Insurance Service (NHIS) cohort sample databases from 2002 to 2010 (Kwon et al., 2015). However, when identifying ONJ, they only relied on the International Classification of Diseases and Related Health Problems – 10th revision (ICD-10) codes (M87.1 and K10.2), and did not consider the case definition of ONJ suggested by the Task Force of the American Society for Bone and Mineral Research (Khosla et al., 2007). Also they did not fully consider confounding factors (such as Paget’s disease, cancer, tooth extraction, and age 15–45 years), which could have influenced the high odds ratio values. Therefore, this study aimed to estimate the age-standardized incidence rate (IR) of ONJ among new users of bisphosphonates among osteoporosis patients older than 50 years in South Korea, and to determine the association between the risk of ONJ and dose intensity of bisphosphonates using a nationwide representative dataset over a 7-year observational period by adjusting for various potential confounding factors.

## Materials and Methods

### Data Source

The dataset used in this study was provided by the Korean NHIS (REQ0000018708). The NHIS established a 7-year cohort spanning 2006–2012 from the National Health Screening Data and the National health insurance claims data. One million men and women were randomly selected from the examinees of the National Health Screening Program between January 1 and December 31, 2006 and merged with the National health insurance claims data of 2006–2012 using the de-identified codes of the examinees. The National Health Screening Program is a mandatory disease prevention program targeting all insured Koreans older than 40 years, and is conducted every other year by the NHIS in order to improve the health of the population and quality of life through early detection, early intervention and disease prevention. The National health insurance claims data pertains to all medical services used by the Korean population, including ICD-10 diagnostic codes, prescribed medicines, surgical histories, as well as demographic information of the population.

### Study Population

In order to identify the impact of dosage of bisphosphonates on the incidence of ONJ, we first identified osteoporotic patients with the ICD-10 codes M80, M81, and M82 from the 7-year cohort. All subjects younger than 50 years were excluded. In addition, those diagnosed with malignant neoplasia (V193, V194) and Paget’s disease (V213) were excluded. These rare or costly diseases are re-confirmed with the specific encoding system (V000) with laboratory data for diagnosis under the Korean NHIS.

**FIGURE 1:**
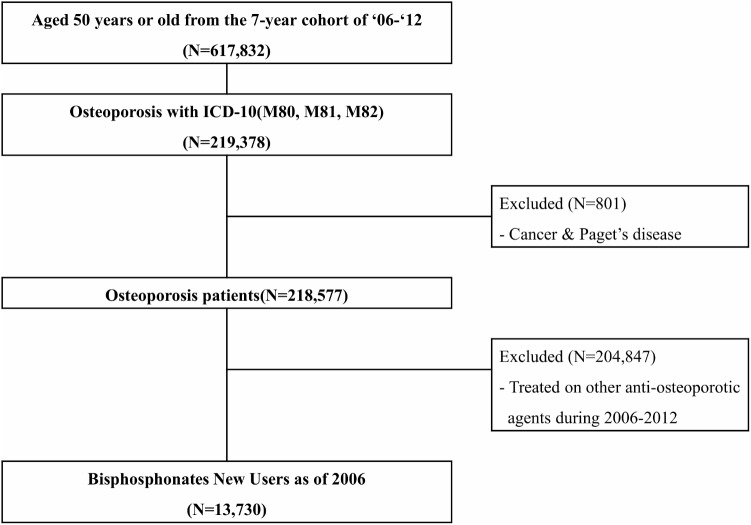
Flow chart for selection of study population, National Health Insurance claim data, Korea, 2006–2012. ICD-10, The International Classification of Diseases and Related Health Problems – 10th revision.

Among the study subjects, we extracted data regarding new users of bisphosphonate as of 2006. Patients who were bisphosphonate naïve in 2006 were selected. In order to avoid any bias due to other anti-osteoporotic agents, subjects who were prescribed calcitonin and selective estrogen receptor modulators more than once during the observational period of 2006–2012 were also excluded. A total of 13,730 subjects were included in the study (**Figure 1**).

### Primary Outcomes

The primary outcome of interest was the ONJ events among bisphosphonate users. ONJ events were first identified by ICD-10 codes (K102, M8710, M8718, M8719, M8780, M8788, and M8789) including osteitis of the jaw, osteonecrosis in multiple sites or unspecified sites due to drugs intake. Of these, those who had continuously treated for partial mandibulectomy, alveoloplasty, and operation of osteomyelitis or bone abscess longer than 8 weeks, during which more than three times of outpatient services only were counted. Subjects were followed until the date of first ONJ event, date of death, or the end of the study period, December 31st 2012.

### Exposure to Bisphosphonates

Bisphosphonates approved in Korea given the observation period were alendronate, risedronate, etidronate, ibandronate, pamidronate, and zoledronate. All dosage forms (i.e., oral or injectable) and formulations (i.e., sustained release for 1 year, 1 month, 1 week, or 1 day) were included. The dose intensity of bisphosphonates was expressed as the number of defined daily doses (DDDs^1^). DDDs are commonly used to standardize different dosages for the comparison of drugs (Whitaker et al., 2012). Thus, we could standardize dosages administrated in a given period of time into DDDs by computing total number of dosages divided by DDDs in each ingredient. For example, the DDD for alendronate is known as 10 mg. For a patient who was prescribed 100 tablets of alendronate 70 mg, the standardized dosages is 700 DDDs (100 × 70 ÷ 10 = 700). This can be interpreted the patient was exposed to alendronate for 700 days. Thus, the number of DDDs can be used as a proxy to the dose-intensity of bisphosphonates. The risk of bisphosphonate-induced ONJ has been known to increase when the duration of therapy exceeds 3–4 years (Ruggiero et al., 2009; Lo et al., 2010). However, currently, Barasch et al. (2011) reported that the risk of ONJ begins within 2 years of treatment and increases dramatically after 2 years (Barasch et al., 2011). Therefore, in this study, we categorized the dose-intensities into three groups: less than 1 year (DDDs < 365.24), 1–2 years (365.24 ≤ DDDs < 730.48) and over 2 years (DDDs ≥ 730.48).

### Covariates

The characteristics of participants such as age, sex, body mass index (BMI), and risk factors of ONJ were included as covariates. Age was treated as a continuous variable, and sex and risk factors were treated as binary variables. BMI was categorized into four groups: <18.5 kg/m^2^, 18.5-22.9 kg/m^2^, 23.0–24.9 kg/m^2^, and ≥25 kg/m^2^. Age, sex, and BMI were determined through history taking at the first prescription of bisphosphonates in 2006. Co-morbidities including rheumatoid arthritis (RA), gingival and periodontal diseases (K05), hypertension (I10-15), hyperlipidemia (E78), and diabetes (E10-14) may also contribute to the risk of developing ONJ (Marx et al., 2005; Badros et al., 2006; Woo et al., 2006). We identified these co-morbidities using ICD-10 codes except RA which was identified according to the V code confirmed with rheumatoid factor blood test. Furthermore, co-medication of glucocorticoid that has been known to be associated with the risk of ONJ was considered a covariate in this study (Marx et al., 2005; Badros et al., 2006). Those treated on the persistent administration of glucocorticoid longer than 60 days during the observation period were regarded as glucocorticoid users.

### Statistical Analysis

For descriptive analysis, the chi-square test was used to compare the difference between the frequency of observed and expected values for significance. Crude and truncated age-standardized IR were estimated to compare the incidence of ONJ according to the dose intensity of bisphosphonates. Crude rate was calculated by dividing the total number of ONJ cases by the total number of person-years of observation and truncated age-standardized IR was estimated by multiplying each crude rate by the weight based on the truncated 2000 United States Standard Population aged 50 years and above and summing the products (Boyle and Parkin, 1991). To examine the impact of dose-intensity of bisphosphonates on the incidence of ONJ, three groups of dose-intensity of bisphosphonates were compared. To do this, we conducted multivariate logistic regression by adjusting for the following demographic and risk factors: age, gender, hypertension, hyperlipidemia, diabetes mellitus, RA, gingival and periodontal disease, and use of glucocorticoids. In all statistical analyses, involving both crude and adjusted odds ratio (OR) with 95% confidence intervals, *P*-values of less than 0.05 were considered statistically significant. All statistical analyses were conducted using SAS version 9.4 (SAS Institute, Cary, NC, United States).

### Ethical Consideration

NHI claim data was provided with anonymous identification number after encoding patients’ personal information to protect their privacy. Thus, patient consent is not required to access the NHI claim data. The study protocol was approved by the Institutional Review Board of Mokwon University, Daejeon, South Korea (IRB number: 2018AA0615).

## Results

### Baseline Characteristics

The total study population was mostly composed of females (92.0%). The mean age of the subjects was 65.2 (7.6 years). As shown in **Table 1**, the demographic features of the study population showed a significant difference for each subgroup. Also, predisposing diseases such as gingivitis or periodontal disease (*P* = 0.025), hyperlipidemia (*P* < 0.001) and RA (*P* < 0.001) demonstrated significantly different distributions among the three groups. The proportion of glucocorticoid users significantly increased with intake of bisphosphonates (*P* < 0.001).

**Table 1:** Basic characteristics of study population stratified by daily defined dose, National Health Insurance claim data, Korea, 2006–2012.

	DDDs < 1 year (*n* = 8,693)	1 ≤ DDDs < 2 years (*n* = 2,167)	DDDs ≥ 2 years (*n* = 2,870)	*P*-value
Sex				
Male, %	860 (9.9%)	113 (5.2%)	124 (8.0%)	<0.001
Female, %	7,833 (90.1%)	2,054 (94.8%)	2,746 (92.0%)	
Age group (years)				
50–59	2,199 (25.3%)	513 (23.7%)	585 (20.4%)	<0.001
60–69	3,688 (42.4%)	1,013 (46.8%)	1,431 (49.9%)	
≥70	2,806 (32.3%)	641 (29.6%)	854 (29.8%)	
BMI (kg/m^2^)				
<18.5	256 (2.9%)	66 (3.0%)	105 (3.7%)	<0.001
18.5–22.9	2,951 (34.0%)	779 (36.0%)	1,072 (37.3%)	
23.0–24.9	2,262 (26.0%)	544 (25.1%)	747 (26.0%)	
≥25.0	3,224 (37.1%)	778 (35.9%)	946 (33.0%)	
Comorbidities				
Gingivitis/periodontal disease, %	492 (5.7%)	155 (7.2%)	182 (6.3%)	0.025
Hypertension, %	3,721 (42.8%)	932 (43.0%)	1,225 (42.7%)	0.973
Hyperlipidemia, %	1,477 (17.0%)	419 (19.3%)	595 (20.7%)	<0.001
Rheumatoid arthritis, %	31 (0.4%)	23 (1.1%)	59 (2.1%)	<0.001
Diabetes, %	1,361 (15.7%)	344 (15.9%)	426 (14.8%)	0.513
Dentoalveolar surgery, %	156 (1.8%)	37 (1.7%)	44 (1.5%)	0.646
Glucocorticoid user, %	31 (0.4%)	12 (0.6%)	52 (1.8%)	<0.001

*BMI, body mass index; DDD, defined daily dose.*

### Incidence Rates of ONJ

**FIGURE 2:**
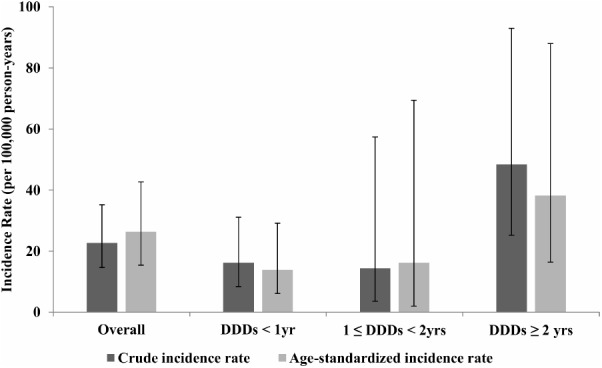
Crude incidence rate and truncated age-standardized incidence rate of osteonecrosis of the jaw stratified by daily defined dose, National Health insurance claim data, Korea, 2006–2012. DDD, defined daily dose; yrs, years.

Overall, 20 patients with ONJ were observed in the study population. Thus, the crude IR of ONJ among the study subjects was estimated as 22.68 per 100,000 person-years (95% confidence interval: 14.64, 35.16) and truncated age-standardized IR was calculated as 26.34 per 100,000 person-years (95% confidence interval: 15.39, 42.67), using the 2000 United States Standard Population aged 50 years and over. **Figure 2** shows that the IR of ONJ differed with the exposure intensity of bisphosphonates. Both crude and truncated age-standardized IR were dramatically increased in the group treated with bisphosphonates for more than 2 years. The truncated age-standardized IR in the group that underwent treatment with bisphosphonates for more than 2 years was calculated as 38.20 (95% confidence interval: 16.43, 88.00), which was substantially higher than the other two groups: 13.85 (95% confidence interval: 6.16, 29.16) for the group treated with bisphosphonates for less than 1 year and 16.19 (95% confidence interval: 1.96, 69.44) for the group treated with bisphosphonates for 1–2 years.

### ONJ Risk With Dose-Intensity of Bisphosphonates

The adjusted ORs for participants with prevalence of ONJ associated with dose-intensity of bisphosphonates are shown in **Table 2**. We conducted multivariate logistic regression analysis using three models in order to effectively control for covariates. Model I shows the adjusted OR of ONJ according to dose-intensity of bisphosphonates by adjusting for sex, age, and BMI. Under the same condition, model II conceived only gingivitis as a risk factor while model III took into account all other co-morbidities including RA, diabetes mellitus, and periodontal diseases.

**Table 2:** Odds ratios for osteonecrosis of the jaw according to daily defined dose and co-morbidities, National Health Insurance claim data, Korea, 2006–2012.

	Model I		Model II		Model III	
	Adjusted OR	95% CI	Adjusted OR	95% CI	Adjusted OR	95% CI
DDDs						
≥2 years	3.68	1.43, 9.46	3.67	1.42, 9.48	3.26	1.23, 8.62
1–2 years	1.02	0.22, 4.75	0.99	0.21, 4.65	0.98	0.20, 4.58
<1 year	1.00	Referent	1.00	Referent	1.00	Referent
Sex						
Female	0.43	0.12, 1.54	0.41	0.11, 1.46	0.41	0.11, 1.47
Male	1.00	Referent	1.00	Referent	1.00	Referent
Age						
	1.12	1.05, 1.19	1.11	1.04, 1.19	1.11	1.04, 1.18
BMI (kg/m^2^)						
≥25.0	2.34	0.21, 3.38	2.32	0.84, 6.38	2.43	0.85, 6.94
23.0-24.9	0.84	0.85, 6.46	0.84	0.21, 3.36	0.85	0.21, 3.48
<22.9	1.00	Referent	1.00	Referent	1.00	Referent
Risk factors						
Periodontal diseases			4.05	1.46, 11.29	4.16	1.48, 11.74
Rheumatoid arthritis					11.51	2.17, 60.95
Hypertension					2.54	0.93, 6.95
Hyperlipidemia					0.62	0.17, 2.20
Diabetes					0.39	0.09, 1.72
Glucocorticoid					3.43	0.93, 6.95

*BMI, body mass index, CI, confidence interval, DDD, defined daily dose, OR, odds ratio.*

Upon adjusting for all potential confounders, participants treated with bisphosphonates for more than 2 years had significantly higher risk of developing ONJ, with an adjusted OR of 3.26 (95% confidence interval: 1.23, 8.62). Aging statistically significantly increased the risk of ONJ while sex and BMI were not statistically significant in this study. Participants with periodontal diseases (OR: 4.16; 95% confidence interval: 1.48, 11.74) and those with RA (OR: 11.51; 95% confidence interval: 2.17, 60.95) had a statistically significantly higher risk of developing ONJ compared to those without these co-morbidities.

## Discussion

This study aimed to investigate the risk of ONJ in accordance with exposure to bisphosphonates administered to Korean osteoporotic patients older than 50 years using a nationally representative dataset. We discovered the truncated age-standardized IR of bisphosphonate-associated ONJ among all subjects with osteoporosis as 20.08 per 100,000 person-years. Various studies have been conducted to estimate the IR of ONJ and similar results were obtained (Lo et al., 2010; Tennis et al., 2012; Lin et al., 2014). According to a retrospective cohort study based on American administrative claim data, the IR of ONJ was estimated as 5 per 100,000 person-years among patients exposed to oral bisphosphonates for osteoporosis (Tennis et al., 2012). Consistent with our results, a study conducted by Lo et al. (2010) in Northern California, reported that the incidence was 28 per 100,000 person-years of oral bisphosphonates treatment (Lo et al., 2010). Lin et al. (2014) also estimated the attributable risk of ONJ associated with alendronate as 77 per 100,000 person-years using the Taiwan National Health Insurance database (Lin et al., 2014).

In addition, through this retrospective cohort study, the IRs of ONJ by dose-intensity were dramatically increased with time to exposure from 13.85 among osteoporosis patients treated for less than a year to 38.20 for treatment of over 2 years. Moreover, this study clearly found that the group treated with bisphosphonates for more than 2 years showed increased odds of developing ONJ compared to the other groups. This was consistent with the findings from a series of studies that the longer the bisphosphonates were taken, the higher the risk of developing ONJ (Bamias et al., 2005; Barasch et al., 2011; Huang et al., 2015). According to a case control study conducted in the United States, the risk of developing ONJ was statistically significantly increased as the duration of bisphosphonate exposure increased. The adjusted OR of patients with 0–2 years, 2–5 years, and more than 5 years of bisphosphonate exposure were 9.9, 39.8, and 38.6, compared to non-bisphosphonate users (Barasch et al., 2011). Huang et al. (2015) based on a Taiwanese National Health Insurance database stated that the risk of ONJ increased significantly with the duration of bisphosphonate use, with a hazards ratio of 4.61 among frequent users, compared to the risk of ONJ among non-bisphosphonate users (Huang et al., 2015). The findings of this study are consistent with previously known time-dependency of bisphosphonate-associated ONJ (Dimopoulos et al., 2006).

In the Korean context, Lee et al. (2013) showed only the epidemiological characteristics of ONJ including incidence ratio among patients undergoing treatment with bisphosphonates, using regional hospital data (Lee et al., 2013). Kwon et al. (2015) demonstrated the significant increase in risk of ONJ among Korean osteoporotic patients who were exposed to bisphosphonate for more than 1.5 cumulative years using NHIS cohort sample data from 2002 to 2010 (adjusted OR: 7.84, 95% CI: 3.97–15.47) (Kwon et al., 2015). However, unlike the study conducted by Kwon et al. (2015), this study was conducted by following up new users of bisphosphonate and identified the ONJ event with a valid definition, and proved that individuals with more than 2 years of bisphosphonate exposure had an increased risk of developing ONJ in South Korea.

This study has several limitations. We could not confirm the diagnosis of ONJ with patient’s medical records since the data we used originated from the National health insurance claims database. On the other hand, we did not distinguish between bisphosphonates in their oral and intravenous forms. Among intravenous bisphosphonate users, a rapid development of ONJ was observed due to the higher and faster accumulation of these drugs (Coskun Benlidayi and Guzel, 2013). Even though we considered dose intensity through DDDs, rapid accumulation of bisphosphonates through intravenous administration was not considered in this study. Thus, stratifying bisphosphonates into oral and intravenous forms may be needed for a more precise estimate in future studies. In addition, adherence to bisphosphonates was not explored in this study, which might affect the outcomes. Considering the adherence to long-term bisphosphonates is known to be poor (Emkey and Ettinger, 2006; Rabenda et al., 2008), the incidence of bisphosphonates-induced ONJ measured based on the claims data could have been underestimated. Despite the above-mentioned limitations, to the best of our knowledge, this is the first study to examine the relationship between ONJ and dose-intensity of bisphosphonates using a national health insurance claims database representative of the South Korean population. The findings clearly support the hypothesis that treatment with bisphosphonates for over 2 years can be a contributing factor to the development of ONJ.

## Conclusion

This study confirmed that Korean osteoporotic patients who had been on bisphosphonates for more than 2 years had an increased risk of ONJ compared to those who were exposed for less than 1 year. The clinical implication of this study is that osteoporosis patients taking bisphosphonates for more than 2 years need to be carefully monitored for possible increased risk of ONJ. In addition, patients with bisphosphonate treatments are strongly recommended to sustain good oral hygiene through the regular dental care.

## Data Availability Statement

This study used NHIS-NSC data (REQ0000018708) made by National Health Insurance Service (NHIS). The data was obtained, following restrictions applies required admission from inquiry committee of NHIS. Requests to access these datasets should be directed to NHIS.

## Author Contributions

S-mJ, SH, and H-YK: development of design and methodology. S-mJ and SH: statistical analysis of data. S-mJ, and H-YK: draft and revised the manuscript.

## Conflict of Interest Statement

The authors declare that the research was conducted in the absence of any commercial or financial relationships that could be construed as a potential conflict of interest.

## Footnotes

1 https://www.whocc.no/atc_ddd_index/
